# 

**Published:** 1965-07

**Authors:** 


### Contents

KENNETH PRIDIE: AN APPRECIATION *A. L. Eyre-Brook* 37

FAMILIAR NEUROSURGICAL PROBLEMS *Douglas G. Phillips* 42

PLACENTAL LOCALIZATION BY ARTERIOGRAPHY
*M. B. Wingate and N. D. O'Connell* 50

CYTOMEGALIC INCLUSION-BODY DISEASE
*Clinico-Pathological Conference* 53

APPOINTMENTS . . . . . . . . . . 57

